# Recurrent respiratory infections in the follow-up of the extremely low birth weight infant

**DOI:** 10.1186/1824-7288-41-S1-A36

**Published:** 2015-09-24

**Authors:** Oliviero Sacco, Michela Silvestri, Giovanni A Rossi

**Affiliations:** 1 Department of Pediatrics, Pulmonary Unit, IstitutoGianninaGaslini, Genoa, Italy

## 

Most of the extremely low birth weight (ELBW) infants, those with a birth weight <1,000 g, are also the youngest of premature newborns, usually with a <27 weeks’ gestational age (wGA). ELBW survival has improved with advancement of neonatal technologies, but survivors may face early and life-long morbidities that includeinfectious respiratory disorders[1]. This increased predisposition is related to a variety of immunological, structural and iatrogenic factors. At the time of term birth, the innate immune response has not fully matured and the adaptive immune system must still develop specificity and memory, completed only in the early childhood [2]. The immaturity of the innate immune systemis more pronounced in preterm infants. The classical, alternative and lectin complement pathways are all reduced in their pathogen-killing abilities and the production and release of soluble antimicrobial proteins and peptidesby leukocytes are deficient. Preterm infants have reduced pool of neutrophils and monocytes and their precursors, compared to term-neonates, and have deficient T cell function with greater proportion of naïve T cells and a low subpopulation of memory T cells. A reduction in lymphocyte subpopulations is still detectable at 8 years of age. There is also a limited production of immunoglobulin (Ig) by the fetus and antigen-specific IgGs are transferred across the placenta from the maternal circulation in large amounts after 32wGA. The lung structures are also structurally and functionally immature in the ELBW. An early complication of extreme prematurity is respiratory distress syndrome caused by surfactant deficiency, with collapse of the alveolar structures, atelectasis edema and decreased lung capacity. Supplemental oxygen and ventilatorysupport may leadto complication, such as air leak syndrome and chronic lung disease of prematurity and increase the risk of persistent damage to the fragile, immature pulmonary structures [3]. Infections are a major contribution to the morbidity and mortality of ELBW infants at any time of the clinical course [4]. The incidence of early-onset infections, due to bacteria from the maternal genital tract but also to nosocomial environmental bacteria and fungi and to airborne viruses, increases in the U.S.A. from <1/1000 live births to 8/1000 in ELBW births. ELBW infants are also at high risk of vaccine-preventable diseases, such as pertussis *and Haemophilus influenzae* and *Corynebacterium diphtheriae* infections: according to the international recommendations, they should receive full doses of the conjugate vaccines at the appropriate chronological age [5].
